# Establishing Pb-203 production from electrodeposited Tl targets at Brookhaven National Laboratory

**DOI:** 10.1186/s41181-025-00403-1

**Published:** 2025-12-10

**Authors:** Wilson Lin, Dmitri G. Medvedev, Cathy S. Cutler, Jasmine Hatcher-Lamarre

**Affiliations:** Brookhaven National Laboratory, Upton, NY 11973 USA

**Keywords:** Pb-203, Theragnostic, BLIP, Thallium electrodeposition, Chromatography, Radiopharmaceutical

## Abstract

**Background:**

Promising developments in Pb-212 radiopharmaceutical therapies have increased demand for Pb-203 diagnostic agents. Building on previous work from various isotope production facilities, this study optimized Pb-203 production from electrodeposited Tl targets at Brookhaven National Laboratory (BNL). The additional supply of Pb-203 may help meet growing preclinical and clinical demands.

**Results:**

Two Tl targets were irradiated at the Brookhaven Linac Isotope Producer facility with 30 ± 1 MeV protons, measured using previously published cross section data. Distribution coefficients for Pb Resin in acetate media were investigated for both Na^+^ and K^+^ cations, where potassium acetate was ~ 4 times more effective at stripping Pb from the Pb Resin. The Tl electrodeposition was optimized to deposit 350 mg of Tl (~ 60 mg/cm^2^) on Au backing in under 6 h. The proposed separation process was completed in < 1.5 h and achieved > 98% and 92 ± 3% recovery of Tl and Pb, respectively, with an overall Tl-Pb separation factor of 6 × 10^5^. The experimentally measured half-life of Pb-203 was 52.4 ± 0.7 h, agreeing with 51.93 ± 0.02 h reported by the National Nuclear Data Center. The radioisotopic purity of the Pb fraction at 24 h post end of bombardment (EOB) from a 24 h irradiation was 66% Pb-203, 28% Pb-201, and 6% Pb-200. Following chemical separation, the Pb-203 produced in this work (21 MBq Pb-203 EOB) achieved apparent molar activities of 10 ± 5 and 0.9 ± 0.5 GBq/µmol for [^203^Pb]Pb-DOTAM and [^203^Pb]Pb-DO3A, respectively, decay corrected to EOB. Data derived from this work suggests BNL can produce > 10’s GBq Pb-203 with > 99% radiochemical and radioisotopic purity from Tl-205 for worldwide distribution.

**Conclusions:**

The production and separation of Pb-203 from natural Tl target material was successfully demonstrated at BNL. Existing methods were adapted and optimized for the facilities at BNL. Results from this work will guide future large-scale Pb-203 production opportunities at BNL for clinical applications.

**Supplementary Information:**

The online version contains supplementary material available at 10.1186/s41181-025-00403-1.

## Background

Targeted Radionuclide Therapy (TRT) with Pb-212 (T_1/2_ = 10.2 h) has demonstrated promising therapeutic efficacies and continues to see increased demand for research and clinical translation (Scaffidi-Muta and Abell 2025; Kokov et al. 2022; Li et al. 2020; Santos et al. 2019; Yong and Brechbiel 2011). Non-invasive pharmacokinetic profiles can be acquired using Pb-203 (T_1/2_ = 51.93 h) tracers as a single photon emission computed tomography surrogate for Pb-212 radiopharmaceuticals. This theragnostic (Aluicio-Sarduy et al. 2019; Brühlmann et al. 2023; Lin et al. 2023; McNeil et al. 2023; Müller et al. 2012; Rösch et al. 2017; Yordanova et al. 2017) approach using chemically matched therapeutic and diagnostic radionuclides has led to growing global demand for Pb-203.

The Brookhaven Linac Isotope Producer (BLIP) at Brookhaven National Laboratory (BNL) is a unique facility capable of irradiating material with protons at incident energies ranging from 66 to 200 MeV (Cannavó et al. 2024; Fitzsimmons et al. 2018; Griswold et al. 2016; NIDC [Internet] 2024; Raparia et al. 2014, 2019; Srivastava 2012). In this regard, Pb-203 can be produced at BLIP by using a suitable incident proton energy (66 MeV) and array of degraders to reach ~ 24 MeV on the Tl target (Qaim et al. 1979; Tárkányi et al. 2013). The produced Pb-203 can then be transferred to the Radionuclide Research and Production Laboratory (RRPL) at BNL and processed in a hot cell suite. Thus, the primary aim of this work was to demonstrate the successful production and purification of Pb-203 at BNL for downstream radiopharmaceutical applications.

There exist several target fabrication and processing methodologies for Pb-203 production, using either isotopically enriched Tl-203 (~ 13 MeV protons) or Tl-205 (~ 24 MeV protons or ~ 31 MeV deuterons) (Internationale Atomenergie-Organisation 2004; Li et al. 2020; McNeil et al. 2021, 2023; Nelson et al. 2023; Saini et al. 2023; Sounalet et al. 2024; Walt and Coetzee pp. 1989). Due to the higher natural abundance of Tl-205 (70.5%), isotopically enriched Tl-203 typically has lower purity (< 99%) and increased costs. Among the different target fabrication techniques, Tl electrodeposition can reduce Tl exposure to personnel and is optimal for target irradiation at BLIP due to its improved thermal characteristics over pressed targets. On the other hand, extraction chromatography with the Pb Resin (Philip Horwitz et al. 1994) is suitable for processing in RRPL and shows the most promise for achieving excellent Pb selectivity over other elements. However, the high affinity of the Pb Resin for Pb increases the elution volume of Pb (McNeil et al. 2023; Nelson et al. 2023; Saini et al. 2023), which warrants further optimization. Additionally, Saini et al. had reported potential inconsistencies in the chemical behavior of Pb-203 when stored in NaOAc (1 M, pH 5.5) for a long period (Saini et al. 2023). Fortunately, since acetate is effective at stripping Pb from the Pb Resin, the authors were able to resolve this by using an additional column loaded with weak cation exchange resin to convert the eluate from NaOAc to 1 M HCl. Although TlNO_3_ precipitation after target dissolution showed promising results to debulk Tl before loading on the Pb Resin (McNeil et al. 2023), this procedure will be difficult to implement for future processing in RRPL hot cells.

This work first investigates the distribution coefficients (K_d_) of Pb and Tl in acetate media for Pb Resin and Chelex 100 resin to optimize the Pb elution profile. Additional batch resin experiments also verified previously measured values for Pb and Tl in HNO_3_ media on the Pb Resin. The Chelex 100 resin was chosen to further purify Pb from Tl and enable acidic media exchange for long-term storage and/or a second purification cycle. Since the methodology in this work was inspired most from Saini et al. (Saini et al. 2023), their separation process was repeated as a baseline for comparisons. The quantities of reagents for Tl electrodeposition were optimized and scaled down to fabricate a single Tl target to assess plating efficiency. Following the successful separation of unirradiated Tl material, two electrodeposited natural Tl samples on Au backing were irradiated at BLIP as a proof of concept for Pb-203 production. The irradiated Tl samples were then processed using the proposed method in this work, and the purified Pb-203 was taken for radiolabeling with commercially available and clinically relevant chelators DOTAM (1,4,7,10-Tetrakis(carbamoylmethyl)-1,4,7,10-tetraazacyclododecane) and DO3A (1,4,7,10-Tetraazacyclododecane-1,4,7-triacetic acid). Finally, this manuscript concludes by discussing potential future work which would greatly benefit Pb-203 production at BNL for clinical radiopharmaceutical applications.

## Methods

All reagents were used without additional purification and acquired from Sigma Aldrich unless otherwise stated. Solutions were diluted using 18 MΩ DI H_2_O (Millipore, Burlington, MA, USA) and/or HNO_3_ (Optima grade, Fisher Scientific).

### Batch resin experiments

K_d_ was measured in triplicate for each media and resin type. Pb Resin (4′,4″(5″)-Di-tert-butyldicyclohexano-18-crown-6 diluted in isodecanol on an inert support, 50–100 µm, Eichrom Technologies Inc. Dry mass 53 ± 2 mg) and Chelex 100 resin (1% cross linkage, 200–400 mesh, Bio-Rad Laboratories. Dry mass 113 ± 7 mg) were placed in 1.5 mL Eppendorf vials and rinsed with > 10 bed volumes (BV) of the respective media. Pb resin was incubated with 0.2, 0.5, 2, and 5 M HNO_3_ and 1 and 2 M Na- or KOAc (both > 99.0%) at pH 5.5. Chelex 100 resin was incubated with 0.5 M and 1.0 M HNO_3_, and 1 and 2 M NaOAc/KOAc at pH 5.5. The resins were in contact with 990 µL of the media spiked with 10 µL of 100 ppm Pb and Tl in 0.05 M HNO_3_ then agitated at room temperature. Aliquots were taken at 15- and 60-min post incubation for analysis with inductively coupled plasma optical emission spectroscopy (ICP-OES, Perkin Elmer Optima 7300 DV). The media used for batch resin experiments had no detectable Tl and Pb.

### Natural Tl targetry

The Tl electrodeposition process on Au substrate was similar to published methods (Internationale Atomenergie-Organisation 2004; Saini et al. 2023), except scaled down to one target mass. Briefly, the electrolyte was prepared by first dissolving 1.8 g NaOH (≥ 99%) in 18 mL DI H_2_O then adding 3.2 g EDTA (99.995% trace metals basis). The solution was buffered using HNO_3_ and/or NaOH until > pH 12 then mixed with 600 µL 50–60% hydrazine hydrate (99.99%) and ~ 100 µL of 30% aqueous BRIJ® 35. Following this, 500–600 mg TlNO_3_ (99.999%) was added to the solution and verified to be > pH 12 upon complete dissolution. The electrolyte was then transferred to the plating cell (Fig. S1).

A platinized titanium mesh anode (0.01″ thick, The Fuel Cell Store) was positioned about 4–5 cm above the Au substrate (0.05 mm thick, 99.95%, Fisher Scientific) with an exposed diameter of ~ 27 mm. A bias voltage of 1.8 V (corresponding to ~ 30 mA) was applied across the plating cell for 5–6 h. After completion, the Tl-Au foil was removed, rinsed with water, then isopropanol, dried, weighed, and stored in a desiccator.

The Al target holder used in this work was similar to previous designs from BNL (Medvedev et al. 2012, 2015). Briefly, a circular Al bolted target holder consisting of two parts held together with 8 Allen screws located at the periphery of the holder was used to isolate the Tl-Au foil for irradiation. A well was machined in the bottom of the holder to accommodate the foil. The foil was sealed from water by a Viton O-ring located in the groove midway between the well and the edge of the holder. The set of degraders for the target array was chosen based on the most optimal energy for Pb-203 production and availability from previously manufactured arrays. The proton irradiation was simulated in FLUKA (version 4–5.0) (Ahdida et al. 2022; Battistoni et al. 2015; Hugo et al. 2024; Lin et al. 2025) using the FLAIR GUI (Donadon et al. 2024) to predict incident proton energy on the Tl material and activation profile. Following the separation process, the undissolved Au foil was used to expose Gafchromic™ films (Ashland, NJ, USA) to ascertain the proton beam spot. The developed Gafchromic™ films were scanned in greyscale and processed using Python. Prior to sample irradiation, the beam position was verified by irradiating plastic film with fiducial markers.

The effective cross section ratios obtained from quantifying radioactive Pb isotopes were used to verify the proton energy based on published cross section data (Tárkányi et al. 2013). Since Tl was expected to degrade the proton energy by ~ 1 MeV, it was approximated as a thin foil. Using the thin foil approximation, and knowing that the Pb isotopes are produced from the same Tl material, the ratio of cross sections can be calculated by:

1 $$\frac{{\sigma }_{1}(E)}{{\sigma }_{0}(E)}=\frac{{A}_{1}(1-{e}^{-{\lambda }_{0}t})}{{A}_{0}(1-{e}^{-{\lambda }_{1}t})}$$

where σ(E) refers to the cross section at some proton energy E, A is the activity at end of bombardment (EOB), λ is the decay constant, t is the irradiation time, and the subscripts denote the respective isotope of interest.

### Dynamic elution studies

The electrodeposited Tl on Au backing (~ 350 mg Tl) was placed in a 20 mL beaker (Tl side facing up) on a hot plate and 5 mL 2 M HNO_3_ was added. The temperature was then set to 80°C and the beaker was periodically agitated using a pair of tongs. After complete dissolution (< 25 min), the solution was transferred to a 25 mL graduated cylinder (McMaster-Carr) and an additional 5 mL 2 M HNO_3_ was used to rinse the beaker. The beaker rinse was transferred to the graduated cylinder, and the volume was recorded (~ 9.8 mL). Then, the solution in the graduated cylinder was transferred to a 50 mL centrifuge tube for easier access, and the graduated cylinder was rinsed with 3 mL 2 M HNO_3_ as part of the rinse step. Two 1 mL solid phase extraction columns were slurry packed with ~ 80 mg Pb Resin (~ 180 mg Chelex 100 resin), washed with > 10 BV DI H_2_O then 1 M HNO_3_, and conditioned with > 10 BV of 2 M HNO_3_ (1 M pH 5.5 NaOAc/KOAc). Depending on the separation process of interest, NaOAc was used for replicating the previously published method by Saini et al. (Saini et al. 2023) and KOAc was used for the proposed method in this work. The flow rate was set at 0.5 mL/min using a peristaltic pump for the entirety of the separation process.

The previously published method for separating Pb from electrodeposited Tl on Au substrate (Saini et al. 2023) was carried out with some modifications to establish a benchmark for the proposed process in this work. Briefly, the Pb Resin column was loaded with the dissolved Tl solution in 2 M HNO_3_, rinsed first with the residual 3 mL of 2 M HNO_3_ solution from the graduated cylinder, and then rinsed with 7 mL of 2 M HNO_3_ followed by 1 mL of 5 M HNO_3_ and DI H_2_O. Pb was then eluted using 6 × 1 mL 1 M pH 5.5 NaOAc and loaded onto the Chelex 100 column. The Chelex 100 column was rinsed with 1 mL DI H_2_O followed by 1 mL 0.01 M HNO_3_, and Pb was eluted using 4 × 300 µL 1 M HNO_3_. The proposed method instead uses 5 × 300 µL 1 M pH 5.5 KOAc to elute Pb from the Pb Resin, with no other modifications except preconditioning the Chelex 100 column with KOAc.

### Lead-203 production from natural Tl and radiolabeling with DOTAM and DO3A

Following promising results from non-radioactive material, electrodeposited Tl foils were test-irradiated in two separate campaigns for future Pb-203 production applications at BLIP (Table 1). The proton energy was degraded from 66 to ~ 30 MeV on target using an existing target array (Table S1). Irradiated samples were then processed using the proposed procedure from above, where the two most concentrated fractions were combined for radiolabeling applications. Aliquots of the purified Pb were taken for analysis with inductively coupled plasma mass spectrometry (ICP-MS, Perkin Elmer ELAN DRC). The second irradiated sample was rinsed briefly with DI H_2_O prior to dissolution due to suspected contamination during the target transfer process.

**Table 1:** Summary of irradiation parameters and measured values from the two Tl irradiation campaigns

Irradiation #	Average beam current (µA)	Irradiation time (h)	Integrated beam current (mC)	Mass of Tl (mg)	Pb-203 EOB yield (MBq)	Pb recovery (%)	Pb-203 apparent molar activity (GBq/µmol)	
							DOTAM	DO3A
1	0.11	21.14	8.4	378	11 ± 1	89 ± 4	3 ± 1	1.1 ± 0.6
2	1.0	4.5	16.2	323	21 ± 1	95 ± 5	10 ± 5	0.9 ± 0.5

The combined Pb fraction was radiolabeled with DOTAM (≥ 95%, Macrocyclics) and DO3A (≥ 95%, Macrocyclics). Apparent molar activity (AMA) for [^203^Pb]Pb-DOTAM and [^203^Pb]Pb-DO3A were determined by titrating 0.37 MBq (10 µCi) Pb-203 with each respective chelator for 30 min at 70°C (buffered using 1 M pH 5.5 NaOAc to pH 5.5, 200 µL final volume). Samples were then analyzed using radio-thin layer chromatography (iTLC-SG, Agilent; pH 5.5, 50 mM EDTA mobile phase). The developed plates were imaged using the AR-2000 Imaging Scanner (Eckert & Ziegler) with WinScan software. Due to the presence of co-produced Pb radioisotopes, the AMA was calculated based on the Pb-203 activity and twice the amount of chelator at 50% radiochemical yield after radiolabeling. The chelator needed for 50% radiochemical yield was estimated from a two-point linear fit based on acquired data below and above 50% yield.

### Sample characterization

Calibration curves for ICP analysis were constructed using certified standards traceable to the National Institute of Standards and Technology (NIST). The following metals were analyzed by ICP-OES, Pb (λ: 220.353, 261.418, and 283.306 nm) and Tl (λ: 276.787 and 351.924 nm); and by ICP-MS, Al, Fe, Cu, Zn, Tl, and Pb.

K_d_ was calculated according to:

2 $${K}_{d}=\left(\frac{{C}_{0}}{{C}_{Aq}}-1\right)\frac{V}{M}$$

where C_0_ is the initial concentration of the metal, C_Aq_ is the concentration of the metal in the solution after resin contact, V is the volume of the solution, and M is the dry mass of the resin. The upper K_d_ limit was set based on a limit of quantification (LOQ) of 30 ppb and the lower K_d_ limit was set based on the maximum relative standard deviation of the readings (15%). Uncertainties in this work are reported as 1 standard deviation about the mean.

Gamma spectrometry was performed using high purity germanium (HPGe) detectors (ORTEC-AMETEK®, Oak Ridge, TN. FWHM at 1173 keV ranged 1.5–1.6 keV) and spectra were acquired with GammaVision software (ORTEC-AMETEK®, v8.10.02). The detectors were calibrated with NIST traceable standards in the same geometry as the sample vials. The calibration efficiency was verified using Eu-152 NIST traceable standard at 344, 779 and 1408 keV. Peak fitting was performed using the CURIE Python library (Morrell 2025).

Half-life measurements were performed by assaying samples at the same fixed geometry over time and fitting the corrected counts to a mono-exponential equation with constant term. The 279.2 (80.9%), 401.3 (3.35%) and 680.5 keV (0.75%) gamma ray energies were used for quantifying Pb-203, and peak counts with signal-to-noise ratio less than 10 were discarded. Samples were assayed again after several months to observe long-lived radionuclides such as Hg-203 (T_1/2_ = 46.6 d, E_γ_ = 279.2 keV, I_γ_ = 81.6%) and Au-195 (T_1/2_ = 186.0 d, E_γ_ = 98.9 keV, I_γ_ = 11.2%).

## Results

### Batch resin studies

**Fig. 1:**
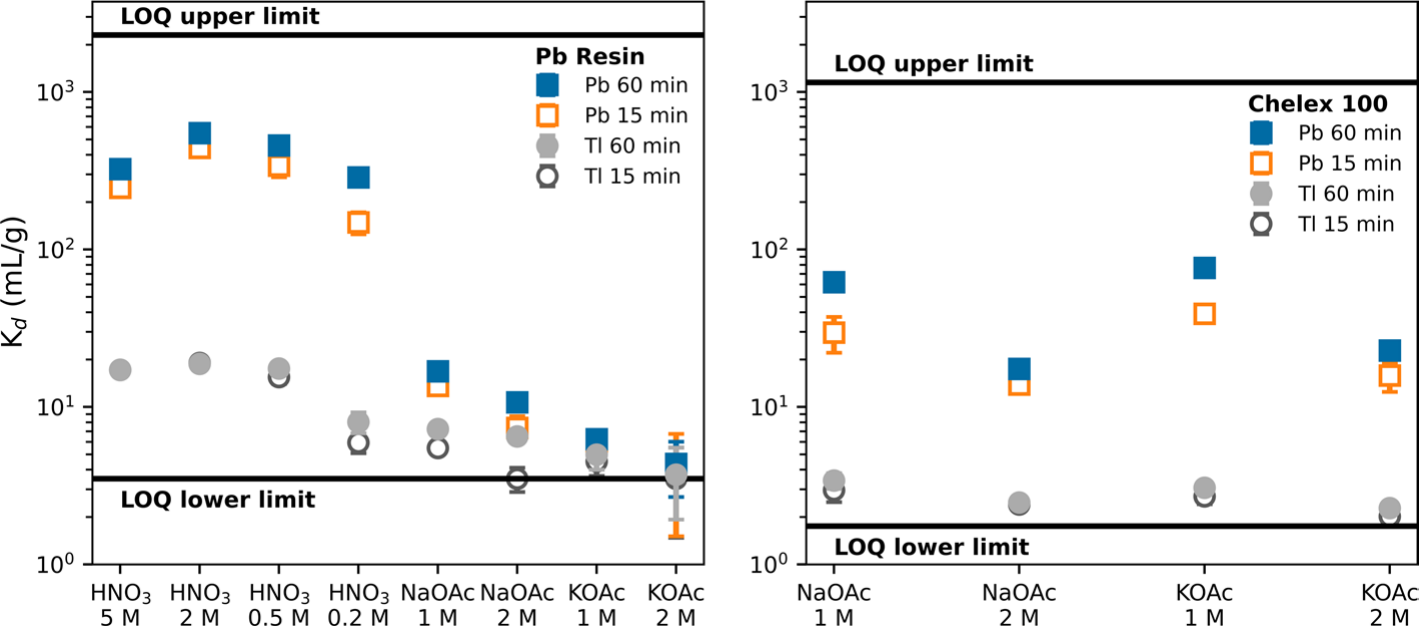
Measured K_d_ values at 15- and 60-min resin contact time for Pb Resin (left) and Chelex 100 resin (right). NaOAc and KOAc refer to sodium and potassium acetate, respectively, both investigated at pH 5.5. The LOQ defines the upper and lower bounds of K_d_ measurements. Chelex 100 K_d_ in 0.5 M and 1.0 M HNO_3_ for both Pb and Tl at all timepoints were below LOQ lower limit and are not shown

To potentially improve upon the previously published separation methods, K_d_ was measured for Pb Resin and Chelex 100 resin in HNO_3_ and acetate media (Fig. 1). The HNO_3_ K_d_ values for Pb and Tl on Pb Resin are comparable to previously published results (Philip Horwitz et al. 1994). Although no published measurements of K_d_ exist for acetate media on the Pb resin, the trend between NaOAc and KOAc agrees with previous observations by Horwitz et al., where KNO_3_ is more efficient than NaNO_3_ at reducing the affinity of Pb on the Pb Resin (Philip Horwitz et al. 1992, 1994). The K_d_ measurements for Chelex 100 resin showed that Pb affinity does not decrease with the presence of K^+^, contrary to the Pb Resin. Also, Pb and Tl both had no observed affinity for Chelex 100 in 0.5 M and 1.0 M HNO_3_. These results suggest that eluting Pb off the Pb resin using KOAc may require less volume than NaOAc, and acidic media exchange is possible with a secondary Chelex 100 column.

### Dynamic elution experiments

The electrodeposition process was completed in 6 h (> 90% material plated) and Tl was completely dissolved in < 25 min at 80°C with 5 mL 2 M HNO_3_. Both separation strategies (in NaOAc and KOAc media, respectively) resulted in similar Pb and Tl recovery and Pb-Tl separation factors. However, 1 M pH 5.5 KOAc was ~ 4 × more efficient than NaOAc at the same concentration and pH for stripping Pb off the Pb resin (Figure S2). These findings correlate well with results from the batch resin studies, where Pb had lower affinity to Pb Resin in KOAc compared to NaOAc. In both cases, bulk Tl can be recovered and added to the electrolyte for electrodeposition. The overall separation process from target dissolution to purification was completed in < 1.5 h.

### Pb-203 targetry and radiochemical processing

**Fig. 2:**
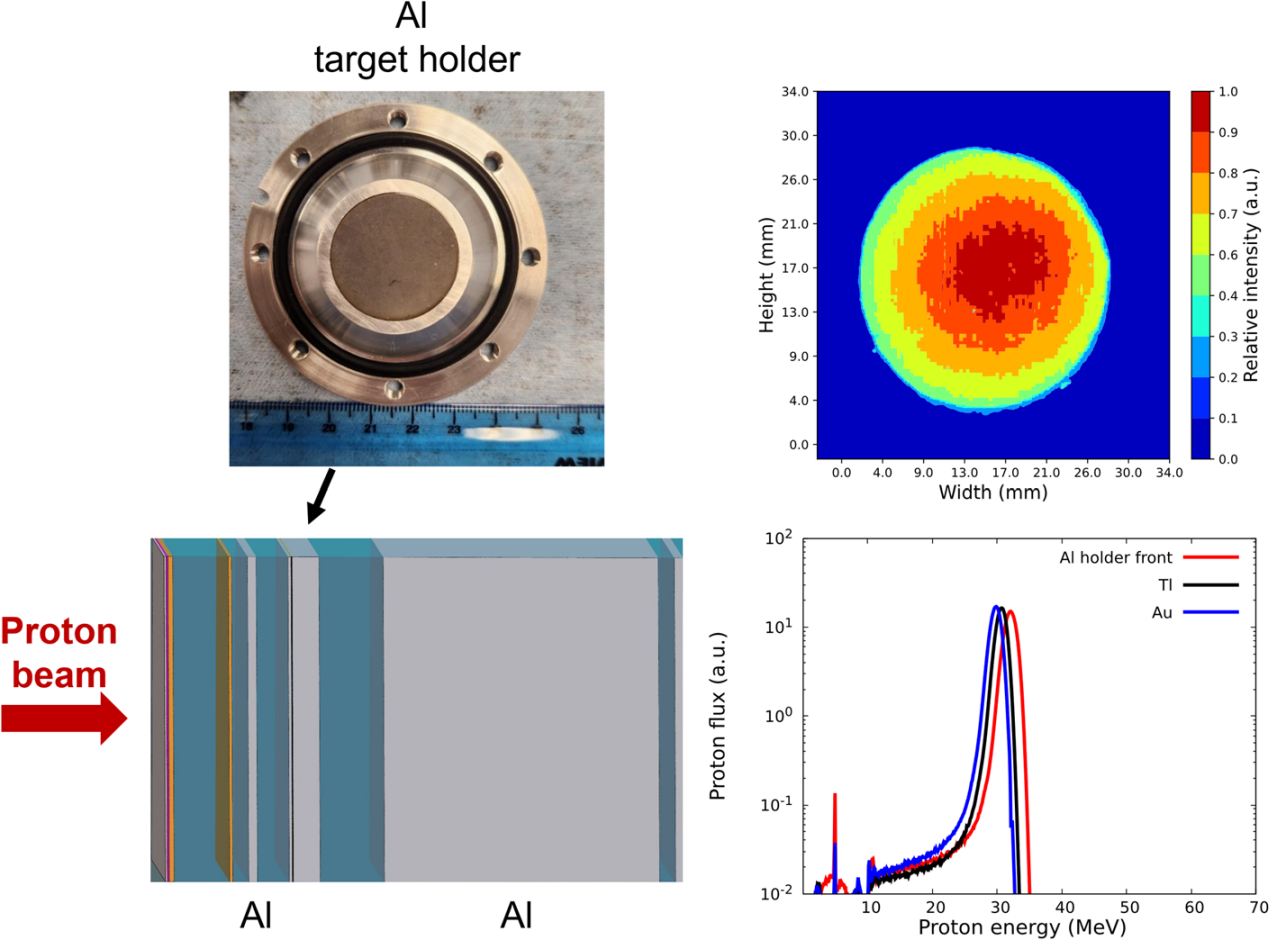
(Top left) A photograph of the electrodeposited Tl foil positioned in the Al target holder. (Bottom left) Schematic representation of the target array, details of the target array are provided in the supplemental information. (Top right) A scan of the Gafchromic™ film after exposure to the irradiated Au foil. (Bottom right) FLUKA simulated proton energy spectrum based on the dedicated degrader array

After optimizing the separation process with KOAc, two electrodeposited Tl samples were mounted on the Al target holder and irradiated at BLIP using a dedicated degrader array. The array was selected to maximize Pb-203 production and minimize radioisotopic impurities based on FLUKA simulations of available array configurations. The FLUKA simulated proton energy in Tl was 30 ± 1 MeV (Fig. 2, bottom right) which agrees with 31 ± 1 MeV (Figure S3) determined from the ratio of experimentally measured cross sections Pb-201/Pb-203 and Pb-200/Pb-203 (Tárkányi et al. 2013). Beam incidence was verified by exposing Gafchromic™ films to the irradiated Au backings, where the proton beam spot was observed to remain within the Tl target diameter (Fig. 2, top right). The Pb-203 physical thin target yield from both irradiation campaigns were comparable when scaled by the thickness (0.36 ± 0.04 MBq/mC-µm). The measured half-life of Pb-203 was 52.4 ± 0.7 h which agrees with the National Nuclear Data Center (NNDC) reported value of 51.93 ± 0.02 h (Fig. S4).

**Fig. 3:**
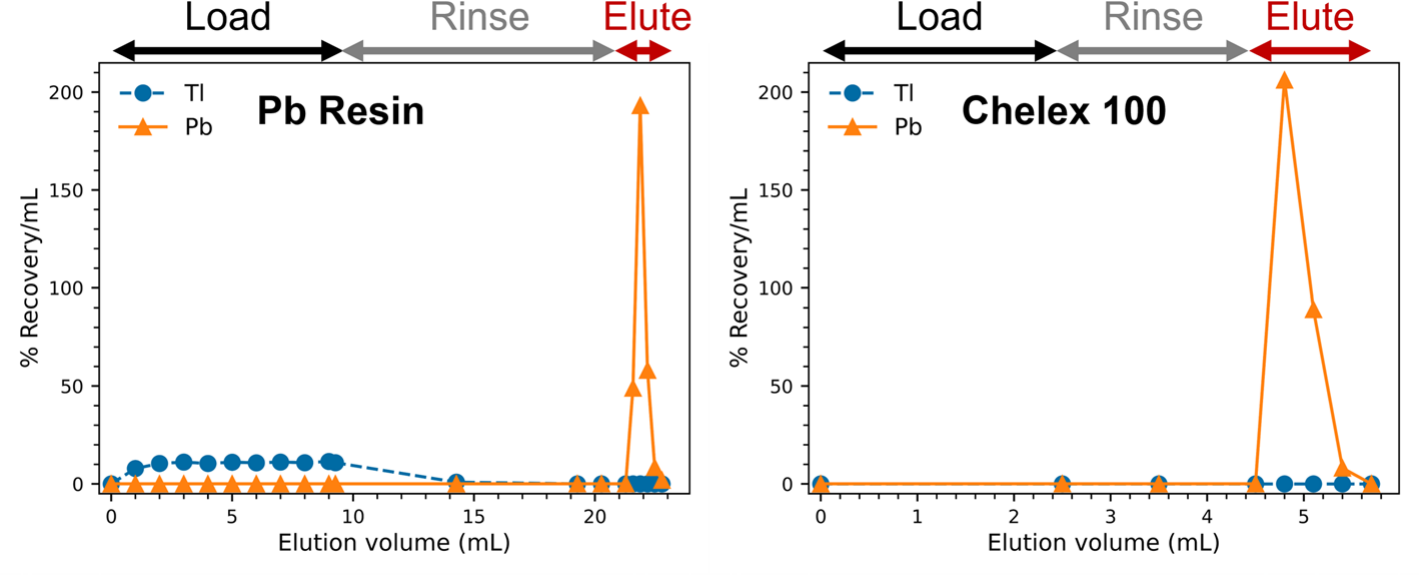
Representative elution profiles of the proposed two-step separation process using Pb Resin (left) and Chelex 100 resin (right). The load, rinse, and elute fractions refer to 2 M HNO_3_ (1 M pH 5.5 KOAc), 2 M HNO_3_ + 5 M HNO_3_ + DI H_2_O (DI H_2_O + 0.01 M HNO_3_), and 1 M pH 5.5 KOAc (1 M HNO_3_) for the Pb resin (Chelex 100 resin)

The separation process for unirradiated and irradiated Tl samples yielded no observable differences, and no physical abnormalities were observed for Tl samples after irradiation. The Tl and Pb recovery were > 98% and 92 ± 3%, respectively, between the two irradiation campaigns, with an overall separation factor 6 × 10^5^ (example elution profiles shown in Fig. 3). Residual activity on both columns accounted for < 3% Pb and < 1% Tl, with negligible Pb breakthrough during the load and rinse. The remaining Pb could be accounted for in pipette tips and transfer vessels. No Au or Hg radioisotopes were observed in the purified Pb fraction. The quantities of natural Pb and Tl in the purified Pb fraction are comparable to previously published results by Saini et al. (Saini et al. 2023) if scaled for the Tl target mass (see Table 2).

**Table 2:** ICP-MS results of the final purified Pb sample from the two irradiated targets

	Al	Fe	Cu	Zn	Tl	Pb
Concentration in ppb (ng)	N.D.^1^	910 ± 180 (540 ± 110)	12 ± 6 (7 ± 4)	N.D.^1^	870 ± 170 (520 ± 100)	940 ± 190 (570 ± 110)

^1^Not detected

### Radiolabeling of [^203^Pb]Pb-DOTAM and [^203^Pb]Pb-DO3A

The two most concentrated Pb fractions from the Chelex 100 column were combined and used for radiolabeling to DOTAM and DO3A (Fig. S5). The [^203^Pb]Pb-DOTAM AMA was measured to be 3 ± 1 and 10 ± 5 GBq/µmol for the first and second irradiation, respectively, and the [^203^Pb]Pb-DO3A AMA was within 1.0 ± 0.5 GBq/µmol for both irradiations. These results indicate that despite the low produced activities (< 22 MBq Pb-203), the radiochemical purity of the produced Pb-203 could be suitable for radiopharmaceutical applications.

## Discussion

Increasing worldwide demand for Pb-203 led us to investigate Pb-203 production at BNL. This work drew inspiration from previous work on Pb-203 production to best suit the capabilities at BLIP and RRPL. Thallium electrodeposition facilitated using Al target holders capable of > 100 µA irradiations at BLIP (Medvedev et al. 2012, 2015). Although not tested in this work, the improved thermal performance of Tl metal previously enabled irradiations at 40 µA (~ 50 µA/cm^2^) by Saini et al. (Saini et al. 2023) and up to 60 µA (~ 76 µA/cm^2^) by Nelson et al. (Nelson et al. 2023). If scaled by the current densities reported previously, the electrodeposited Tl samples in this work should withstand > 100 µA (> 17 µA/cm^2^) irradiations at BLIP (Raparia et al. 2019). Based on the experimental results from this work, the Tl targets withstood ~ 2 µA of peak beam current using a focused beam which implies that a raster pattern (Raparia et al. 2019) will enable irradiations with at least 10 µA. Such an increase in beam current would commensurately increase Pb-203 production. Following irradiation at BLIP, radiochemical processing in RRPL for large-scale isotope production is generally performed in hot cells and operated through mechanical manipulators. This work employed a two-step processing method derived from Saini et al. (Saini et al. 2023) using only chromatographic separation strategies. Although the method by Nelson et al. (Nelson et al. 2023) was also based on column chromatography and achieved successful Pb-Tl separation, the overall processing time was several hours longer (see Table 3).

**Table 3:** Summary of Saini et al. and Nelson et al.’s results and this work

Author (reference)	Tl mass (mg)	Total processing time	Total volume (mL)	Final Tl recovery (%)	Final Pb recovery (%)
Saini et al. 2023	~ 100	< 1.5 h	~ 28	Unspecified	92.3 ± 3.5
Nelson et al. 2023	~ 250	< 4 h	~ 135	92	85
This work	~ 350	< 1.5 h	~ 28	> 98	92 ± 3

The large elution volume required to strip Pb off the Pb Resin reported in previous works (McNeil et al. 2021, 2023; Nelson et al. 2023; Saini et al. 2023) was identified as a potential area of improvement. In characterizing and developing the Pb Resin, Horwitz et al. showed that weak chelating agents such as OAc could be used to remove Pb from Pb Resin (Philip Horwitz et al. 1992, 1994). For radiolabeling applications, NaOAc and NH_4_OAc may also have been chosen as stripping agents for convenience due to their ubiquitous use as a pH buffer and low affinity for Pb relative to other chelators. However, despite widespread use of Na/NH_4_OAc as stripping agents for Pb, there is a lack of K_d_ evaluations in OAc media with Pb Resin. Horwitz et al. reported that KNO_3_ reduced the affinity of Pb on Pb Resin in contrast to NaNO_3_ and Ca(NO_3_)_2_ under otherwise identical conditions (Philip Horwitz et al. 1994). Similar experiments for Sr on Sr Resin suggest the presence of NH_4_^+^ can also reduce the Pb Resin’s affinity for Pb but with less potency than K^+^ (Philip Horwitz et al. 1992). The mechanism of action for the preference of K^+^ over Na^+^/NH_4_^+^ in Pb Resin (a 18-crown-6 derivative) is likely due to a size match selectivity combined with the chelation effect from oxygen donors (Bradshaw and Izatt 1997; Hancock 1992, 1994). Thus, we hypothesized that KOAc may be a more effective media to elute Pb from Pb Resin and evaluated this idea first through batch resin experiments. Since the two-step separation process was already adopted to reconstitute Pb in acidic media without OAc for long-term storage, the residual K^+^ ions can be removed simultaneously with OAc. In comparison to eluting with NaOAc (~ 6 mL), KOAc elutes Pb in ~ 4 times less volume from Pb Resin (~ 1.5 mL). Also, the overall separation process took < 1.5 h, does not require precipitation to de-bulk Tl, and results in labile Pb-203 for at least 3 days post purification in dilute acidic media.

The mass of electrodeposited Tl used in this work (320–380 mg, 50–60 µm or 60–70 mg/cm^2^) was several times heavier than by Saini et al. (~ 100 mg, ~ 100 µm or 76–114 mg/cm^2^) but comparable to McNeil et al. (~ 330 mg, ~ 350 µm or ~ 400 mg/cm^2^) and Nelson et al. (~ 250 mg, 270 µm or 320 mg/cm^2^) (McNeil et al. 2021, 2023; Nelson et al. 2023; Saini et al. 2023). However, due to safety envelope constraints at BNL, the activity of Pb-203 produced in this work (0.02 GBq) was substantially less than the other facilities (0.2–5 GBq). Despite this, radiolabeling experiments with the purified Pb-203 product demonstrate that the sample already meets the current specifications of > 1.85 GBq/µmol Pb molar activity and could be used for research applications. Further improvements to the molar activity can be achieved through full-scale irradiations at BLIP, which would yield GBq quantities of Pb-203 without changing the Tl mass and separation procedure. The major contaminant detected in the purified Pb-203 sample was natural Pb, which can likely be reduced with additional target recycling. If necessary, other trace contaminants can be removed with an additional Pb Resin-Chelex 100 purification cycle without appreciable loss to decay (< 0.5 h) and processing. This second cycle can be performed immediately using the eluted < 1 mL 1 M HNO_3_ Pb solution. Alternatively, if HCl is preferred instead of HNO_3_, the method can also be altered to elute Pb off the Chelex 100 column with 1 M HCl. Nevertheless, the purified Pb sample in this work can meet current (radio)chemical purity specifications for clinical applications, especially if ~ GBq Pb-203 is produced.

**Fig. 4:**
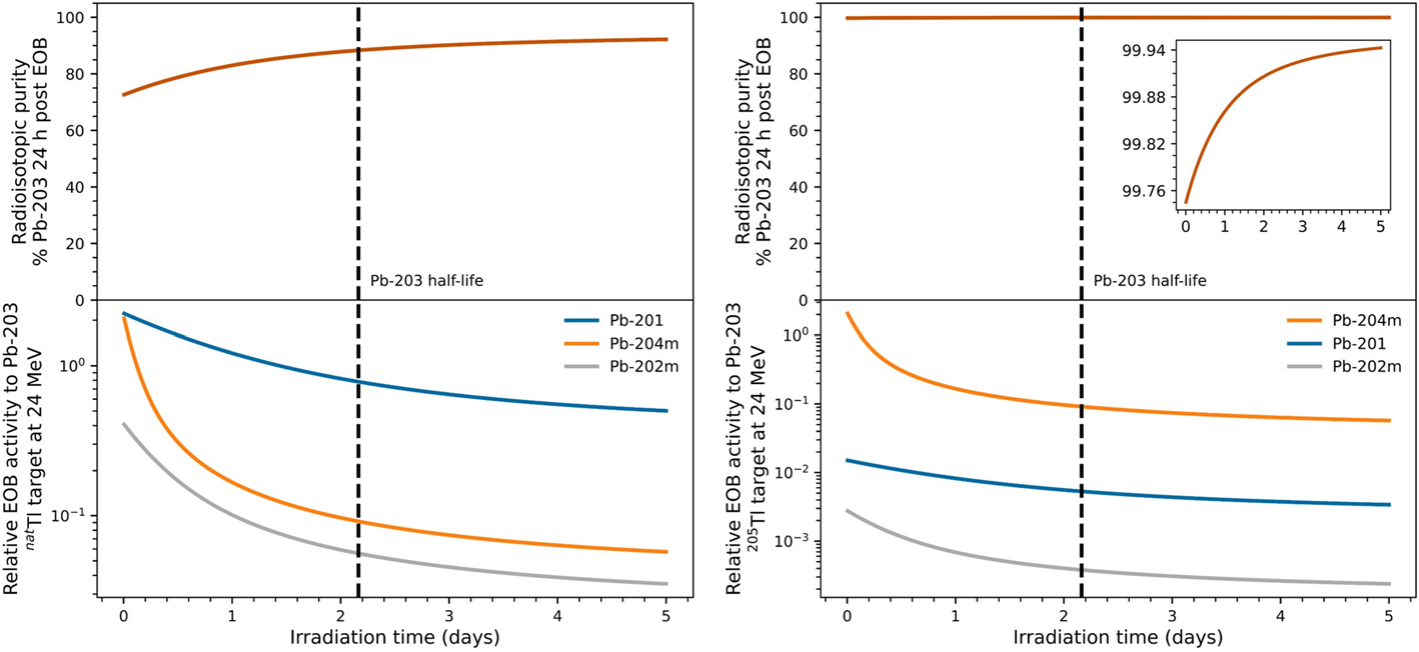
The expected radioisotopic purity (top) of Pb-203 at 24 h post EOB for natural (left) and 99.8% isotopically enriched Tl-205 (right) target material for a given irradiation duration. The inset plot in the top right was provided to better visualize the radioisotopic purity at > 99%. Due to differences in the energy threshold and half-life, the relative EOB activity is dominated by different Pb radioisotopes (bottom). After 24 h post EOB, Pb-201 will remain as the main radioisotopic impurity for both target materials

While the radioisotopic purity for Pb-203 was only 66% at 24 h post EOB following a 24 h irradiation using natural Tl (Fig. S6), isotopically enriched Tl-205 (> 99.8%) can be acquired for ~ $3/mg to increase the radioisotopic purity to > 99% (Fig. 4) with the same irradiation parameters. For reference, the threshold energy of Pb-202m (T_1/2_ = 3.54 h) and Pb-201 (T_1/2_ = 9.33 h) is 25 MeV and 32 MeV, respectively, for proton induced reactions on Tl-205. Operating costs associated with Tl-205 material can be neglected based on quantitative recovery of Tl in the separation process. The decay duration of 24 h post EOB serves as a practical measure of time for target transfer, chemical processing, and Pb-203 distribution. Due to programmatic requirements at BNL, the target array used in this work was built from existing hardware that was most suitable for Pb-203 production. However, the target array can be further optimized for Pb-203 production to achieve ~ 24 MeV on target by increasing the thickness of the first Al degrader (Fig. S7). Although this work did not irradiate isotopically enriched Tl-205, Saini et al. (2023) and Nelson et al. (2023) had previously produced Pb-203 with > 99% radioisotopic purity from Tl-205 material using protons with the proposed incident energy (~ 24 MeV). Extrapolating results from this work to a full-scale production, a 50 µm thick Tl-205 target irradiated with 24 MeV protons at 50 µA for 24 h will produce 13 GBq (0.34 Ci) Pb-203 with > 99% radiochemical and radioisotopic purity at 24 h post EOB. Consequently, using Tl-205 as the target material during full-scale production at BNL and adopting the proposed separation process presented in this work will enable Pb-203 distribution for clinical applications.

## Conclusion

This work establishes Pb-203 production and processing at BNL and demonstrates the potential for BNL to be a global supplier of Pb-203. Two electrodeposited Tl samples were irradiated at BLIP as proof of concept for large-scale Pb-203 production. The measured average energy in Tl was 30 ± 1 MeV based on the ratio of cross sections, and the physical thin target yield of Pb-203 scaled by Tl thickness was 0.36 ± 0.04 MBq/mC-µm. Batch resin experiments optimized dynamic elution studies, where substitution of K^+^ for Na^+^ in OAc media enabled ~ 4 × more efficient Pb stripping from Pb Resin. Electrodeposition of Tl targets from TlNO_3_ was completed in < 6 h, and the separation process recovered > 98% Tl and 92 ± 3% Pb with a separation factor of 6 × 10^5^. The measured half-life of Pb-203 was 52.4 ± 0.7 h, agreeing with NNDC’s reported value of 51.93 ± 0.02 h. The AMA of [^203^Pb]Pb-DOTAM and [^203^Pb]Pb-DO3A was 10 ± 5 and 0.9 ± 0.5 GBq/µmol, respectively, at EOB with only 16.2 mC on target (21 MBq Pb-203 EOB). Although the radioisotopic purity of Pb-203 in this work was 66% by activity at 24 h post EOB following a 24 h irradiation, using isotopically enriched Tl-205 could achieve > 99% radioisotopic purity without any other modifications. Further adjustments to the first Al degrader in the target array and irradiation duration can achieve improvements in both production yield and radioisotopic purity. Using results derived from this work, > 10’s GBq Pb-203 can be distributed from BNL for clinical applications.

## Supplementary Information

Below is the link to the electronic supplementary material.

Supplementary Material 1

## Data Availability

The datasets used and/or analyzed during the current study are available from the corresponding author on reasonable request.

## Abbreviations

AMA: Apparent molar activity
BLIP: Brookhaven Linac Isotope Producer
BNL: Brookhaven National Laboratory
BV: Bed volume
DO3A: 1,4,7,10-Tetraazacyclododecane-1,4,7-triacetic acid
DOTAM: 1,4,7,10-Tetrakis(carbamoylmethyl)-1,4,7,10-tetraazacyclododecane
EOB: End of bombardment
HPGe: High purity germanium
ICP-MS: Inductively coupled plasma mass spectrometry
ICP-OES: Inductively coupled plasma optical emission spectroscopy
LOQ: Limit of quantification
NIST: National Institute of Standards and Technology
NNDC: National Nuclear Data Center
RRPL: Radionuclide Research and Production Laboratory
TRT: Targeted radionuclide therapy
