# Supplementary material for: Establishing Pb-203 production from electrodeposited Tl targets at Brookhaven National Laboratory
Source: EJNMMI Radiopharm Chem. 2025 Dec 10;10:78. doi: 10.1186/s41181-025-00403-1 (PMC12696209; doi:10.1186/s41181-025-00403-1)
Supplement: Supplementary file 1 — Supplementary Material 1 [file 41181_2025_403_MOESM1_ESM.docx]

**Supplemental information**


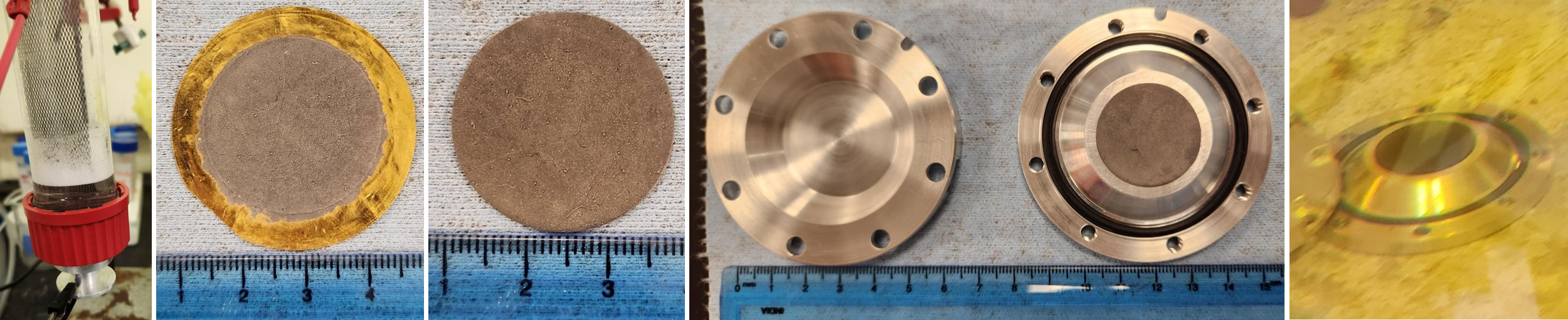


Figure S1. From left to right: the electroplating cell, electrodeposited Tl on Au foil, Tl-Au sample after removing excess Au, Al target holder with Tl-Au sample mounted, and the irradiated Tl-Au foil as seen through the hot cell window at BLIP. No physical differences were observed before and after irradiation.


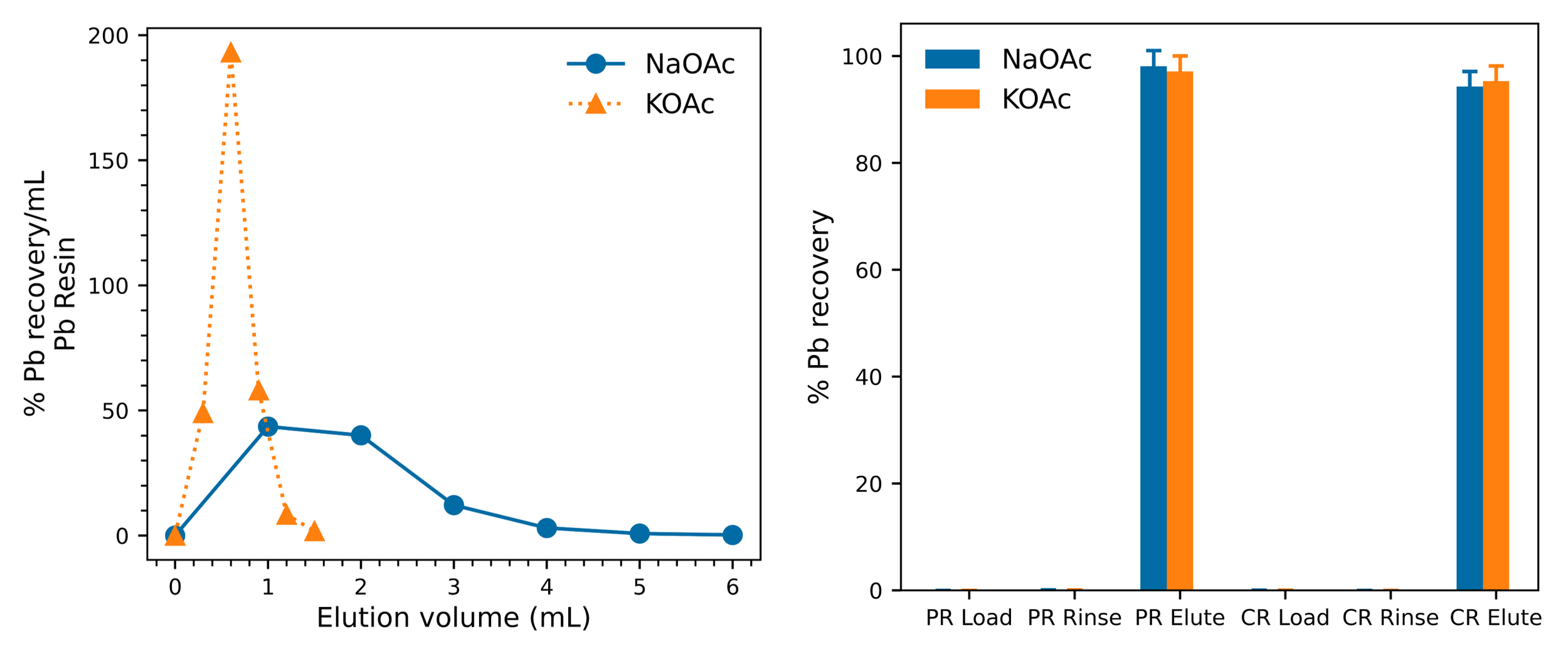


Figure S2. (Left) The elution profile of Pb from the Pb resin using KOAc (orange dotted lines, triangle) shows that KOAc is more effective than NaOAc (blue solid lines, circle) at stripping Pb off the resin (both 1 M, pH5.5). (Right) The Pb in KOAc media was well retained by Chelex 100 resin and had negligible breakthrough, leading to quantitative recovery of Pb. Both separation strategies resulted in similar Pb recovery. “PR” refers to the Pb Resin and “CR” refers to the Chelex 100 resin. “Load”, “Rinse” and “Elute” refer to the entirety of the collected load, rinse and elution fraction, respectively.


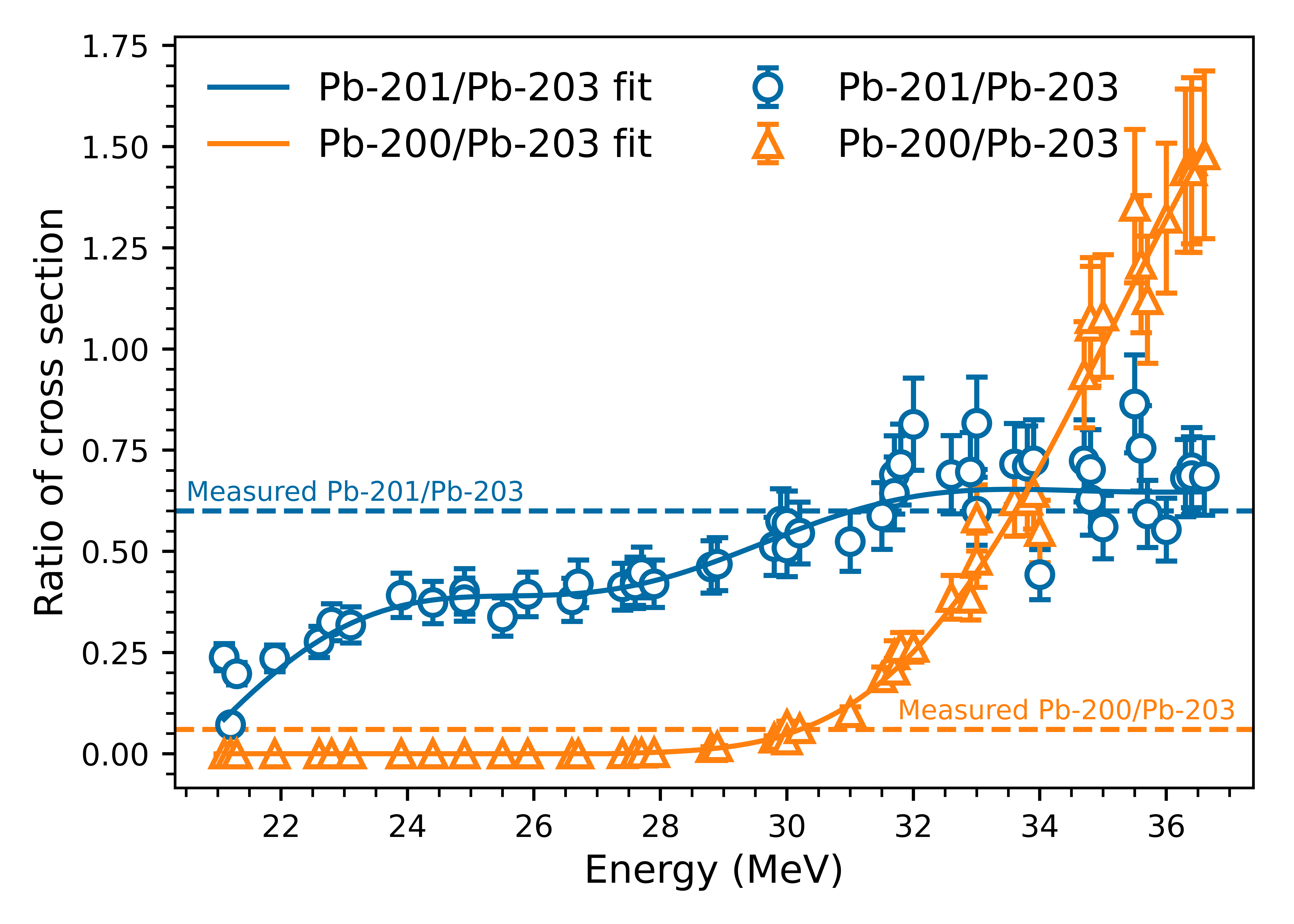


Figure S3. Previously measured Pb-201/Pb-203 (blue circle) and Pb-200/Pb-203 (orange triangle) nuclear cross section ratios from (14) were fit using Gaussian Process Regression (solid lines). Effective cross section ratios measured in this work (dotted horizontal lines) were used to verify the proton energy for the Tl sample. The average cross section ratios were 0.58±0.03 and 0.07±0.01 for Pb-201/Pb-203 and Pb-200/Pb-203, respectively.


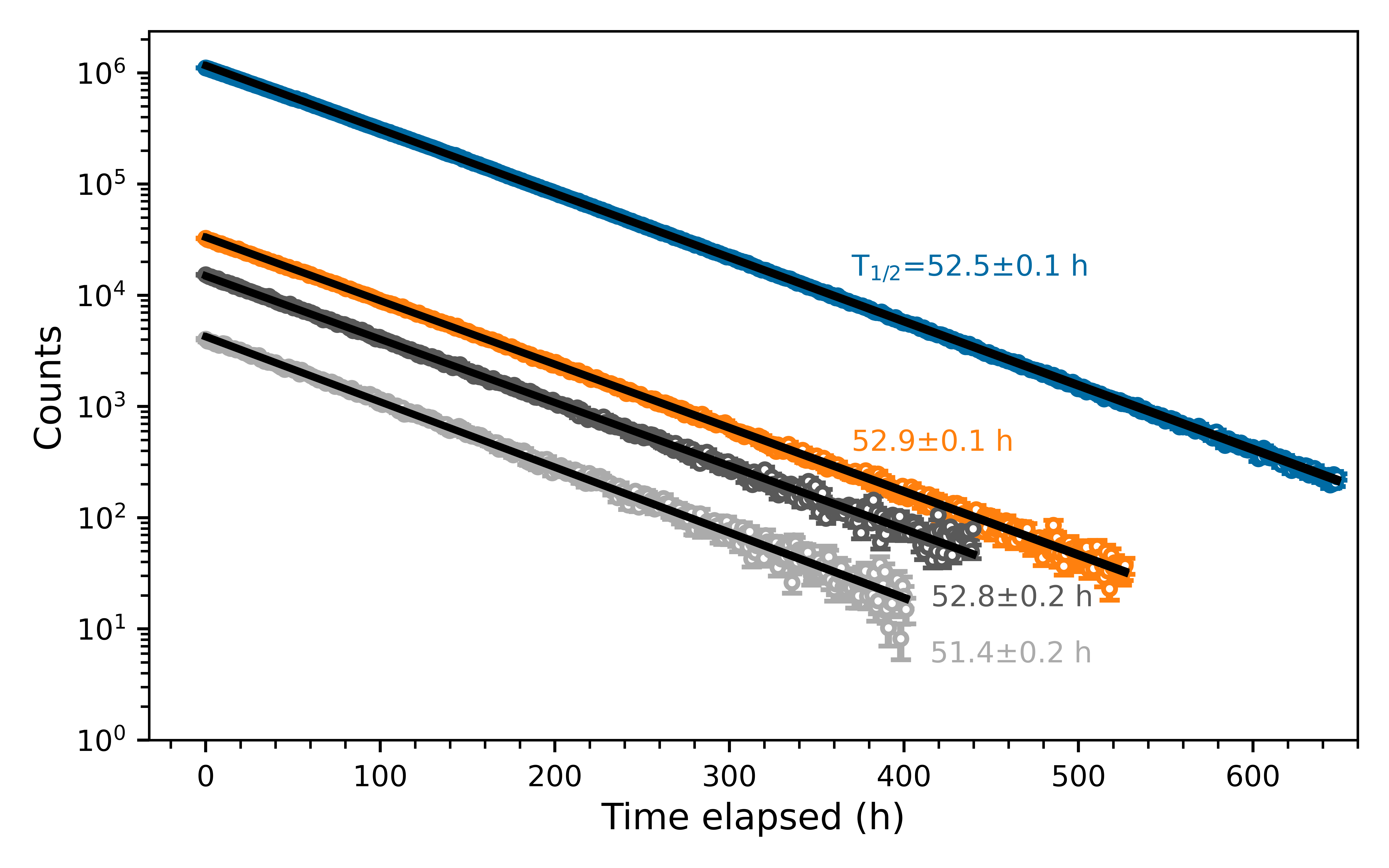
Figure S4. The half-life of Pb-203 was determined by continuously counting several samples at fixed geometry. The samples used for radiolabeling were combined into one fraction to improve counting statistics. The measured half-life of Pb-203 was 52.4±0.7 h.


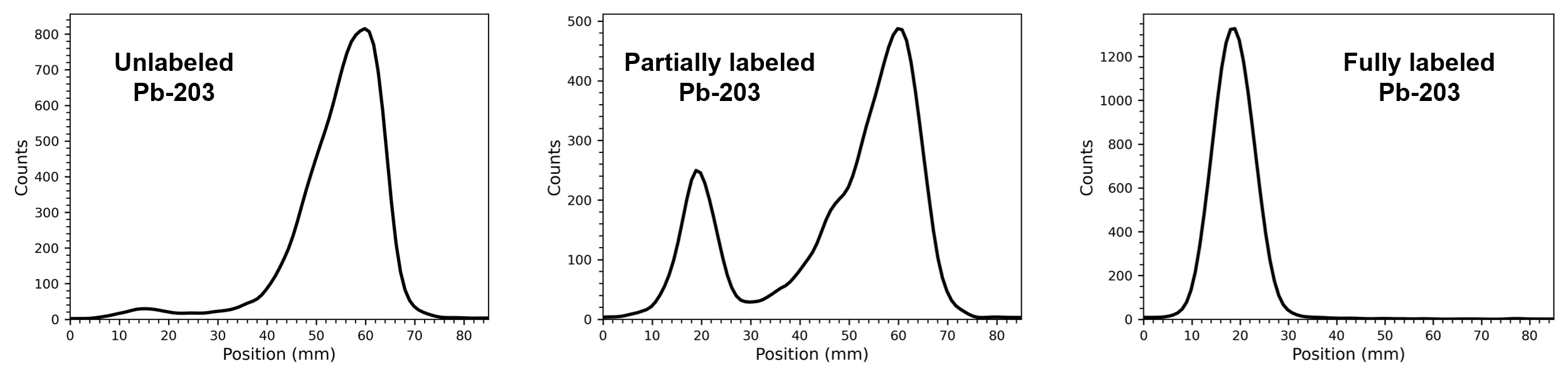


Figure S5. Representative radio-TLC chromatograms of the unlabeled (left, R_f_=0.7), partially labeled (middle) and fully labeled (right, R_f_=0.2) [^203^Pb]Pb-DOTAM complex. The [^203^Pb]Pb-DO3A complex showed similar radio-TLC chromatograms. These chromatograms were filtered using a smoothing spline algorithm.


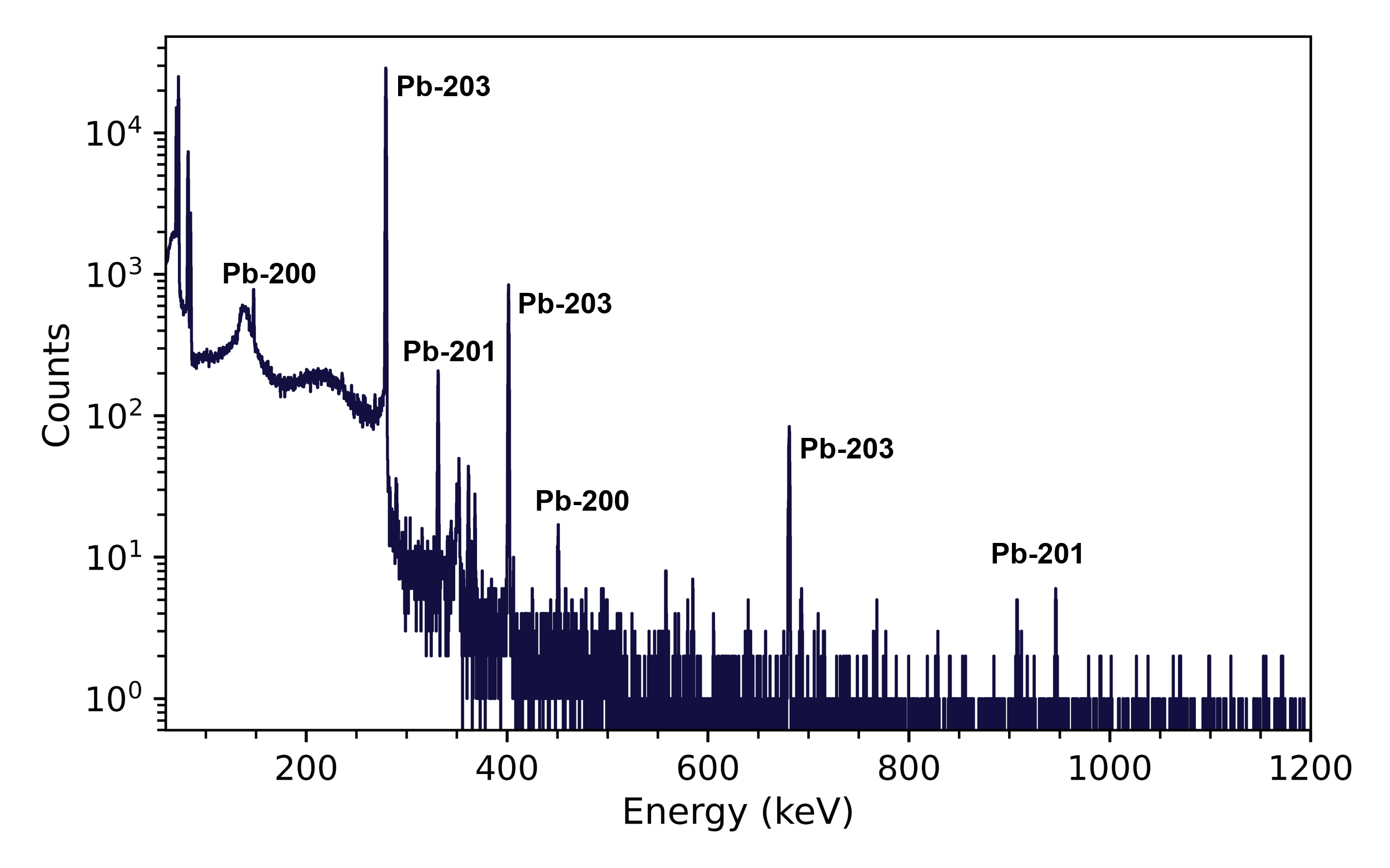


Figure S6. HPGe spectrum of the purified Pb-203 approximately 2 h post purification.


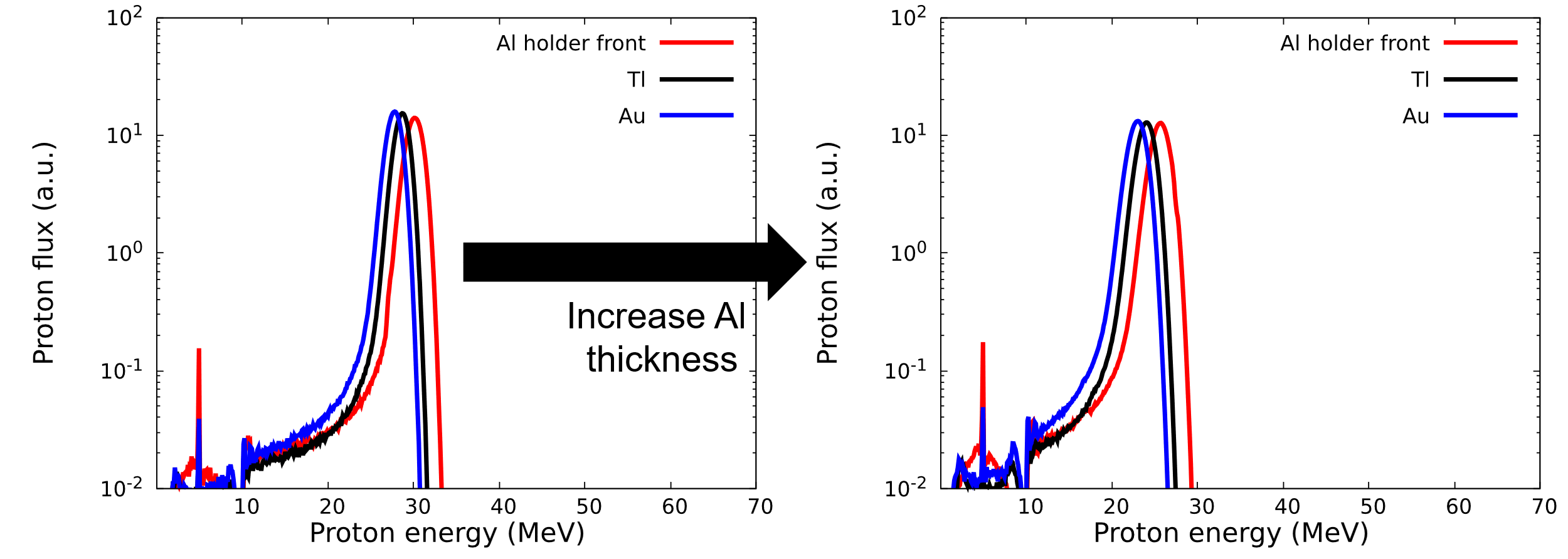


Figure S7. Increasing the first Al degrader thickness to 2.50 mm changes the FLUKA simulated proton energy on Tl from 30±1 MeV (left) to 24±1 MeV (right).

Table S1. The Tl target array configuration simulated in FLUKA.

| Material | Thickness of material (cm) |
| --- | --- |
| Water | 1.00E-03 |
| Beryllium | 3.05E-02 |
| AlBeMet | 3.05E-02 |
| Stainless steel | 7.87E-02 |
| Water | 8.69E-01 |
| Stainless steel | 5.08E-02 |
| Water | 2.54E-01 |
| Aluminum | 1.42E-01 |
| Water | 5.08E-01 |
| Aluminum | 5.08E-02 |
| Thallium | 5.16E-03 |
| Gold | 5.08E-03 |
| Aluminum | 4.32E-01 |
| Water | 1.03E+00 |
| Aluminum | 4.41E+00 |
| Water | 5.08E-01 |
| Aluminum | 1.42E-01 |
